# Editorial: Target organ damage in Fabry disease

**DOI:** 10.3389/fcvm.2025.1651117

**Published:** 2025-07-08

**Authors:** Guido Iaccarino, Francesca Graziani

**Affiliations:** ^1^ Department of Clinical Medicine and Surgery, Federico II University, Napoli, Italy; ^2^ Department of Cardiovascular Sciences, Agostino Gemelli University Polyclinic (IRCCS), Rome, Italy

**Keywords:** Fabry disease, genetics, treatment, mechanisms, target organ damage

**Editorial on the Research Topic**
Target organ damage in Fabry disease

## Introduction

The body of research dedicated to Fabry Disease (FD) has grown significantly in the last 25 years, concurrent to the availability of therapies for the disease. Currently, consolidate therapies include enzyme replacement and chaperone therapy. Experimental therapeutics, such as gene therapy and substrate supplementation, are in the pipeline to be deployed in the clinical realm.

With greater awareness among clinicians and researchers, the number of diagnosed cases has risen, along with the identification of putative pathogenic mutations in the GLA gene (encoding GalA). To date, more than 1,500 such mutations have been reported, and the number continues to grow. Some mutations have been shown to have a clear impact on the disease, while others remain of uncertain clinical relevance (1).

As more clinical cases are studied, the heterogeneity in the disease's clinical presentation has become increasingly apparent. The relationship between genotype and phenotype appears to be less straightforward than initially assumed, and target organs might be affected differently despite the presence of similar or even the very same mutation, in particular with variants of unknown significance. Notably, since the GLA gene is located on the X chromosome, significant sex-based differences in clinical manifestation have been observed (2). Males typically exhibit a more severe disease phenotype, with greater target organ involvement and shorter survival, while females often present with a milder clinical course and longer life expectancy (3). This calls for different management strategies between male and female Fabry patients, as pointed out by Tuttolomondo et al.

**Figure 1:**
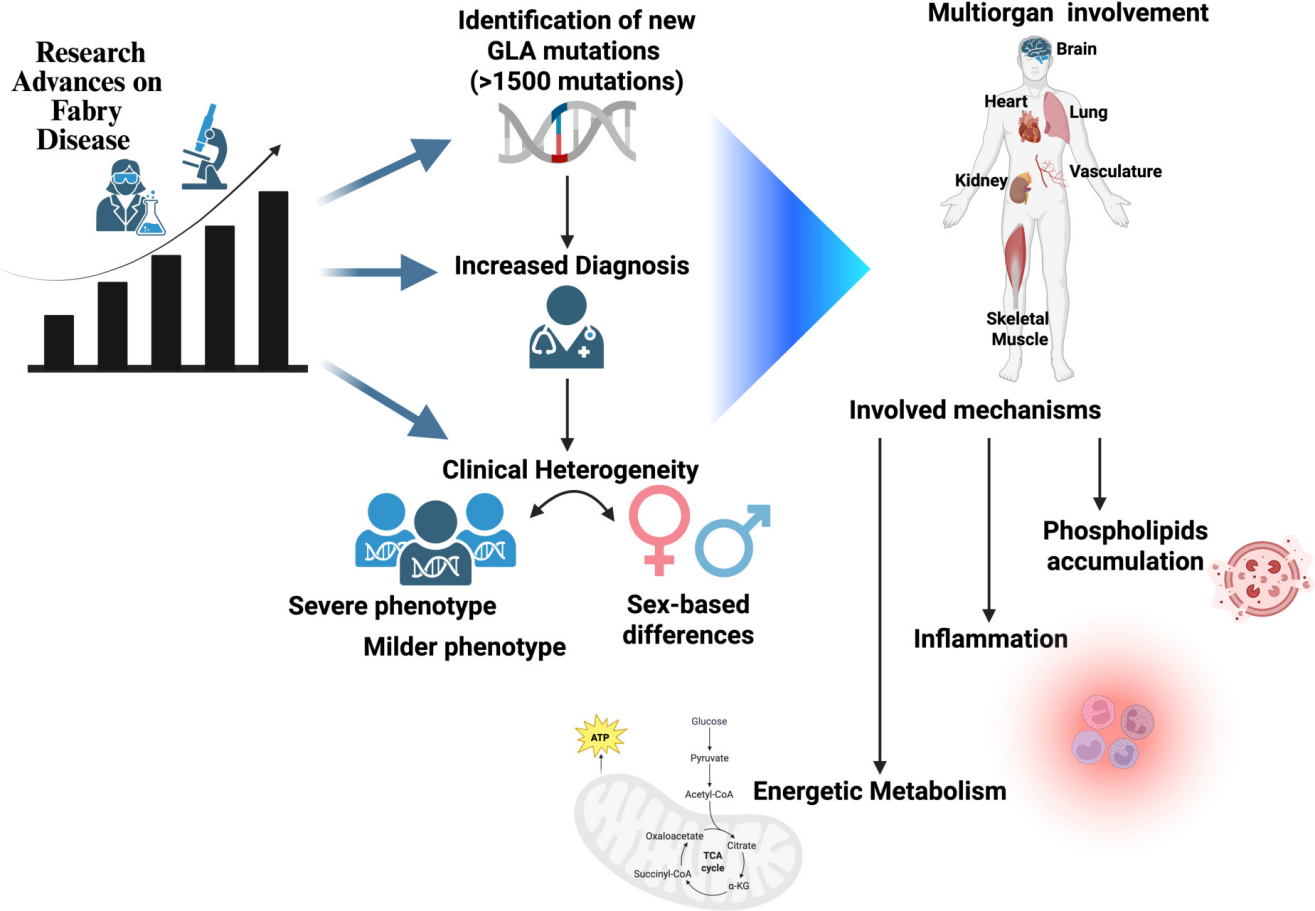
An increased volume of papers in recent years has revealed the complexity of Fabry disease, which presents with intermediate phenotypes, different involvment of target organs, and a non-univocal relationship between genotype and phenotype. Sex is one determinant of such variability; novel mechanisms of disease can also be considered as possible modifiers.

All of the above suggests that the mechanisms of FD might be more complicated than originally thought. In a classical vision, the damage of target organs such as the heart, kidney, brain, bowels, and vasculature is due to the intracellular accumulation of globotriaosylceramide (Gb3), which leads to cellular dysfunction and therefore loss of organ function (4). Such a vision, though, implies an important GB3 intracellular accumulation that in some cases is barely present or even missing entirely. Although lysosomal dysfunction can be considered the beginning of cellular dysfunction, other mechanisms need to be taken into account that might accelerate or delay the onset of the target organ damage associated with the disease (5) (Figure 1).

In this Research Topic dedicated to the Target Organ Damage (TOD) in FD, we focused on the current interpretation of clinical manifestation.

Twelve papers, with over 100 authors and more than 18,000 total views at the time this editorial is written, confirm the renewed interest in this condition.

## The heart

The heart is the target organ of FD that has been most often investigated. In this RT, it is clear that the cardiac ultrasounds (cardiac US) remains the easiest and most accessible way to assess cardiac damage in FD. All cardiac structures, such as the left ventricle and the left atrium, the aorta, the right sections, and the heart valves, can be affected by morphological and functional abnormalities. Conte et al. state that standard echocardiography has a crucial role in the characterization of FD cardiomyopathy and provide a comprehensive review on the topic. Furthermore, echocardiographic evaluation is an essential imaging method to support the physician in follow-up and risk stratification. Spinelli et al. suggest that techniques such as tissue Doppler imaging and speckle-tracking echocardiography (STE) allow detection of subclinical changes in left ventricular (LV) systolic and diastolic function, particularly reductions in global longitudinal strain and circumferential strain gradients. These techniques are also valuable for evaluating right atrial and ventricular involvement, often preceding hypertrophy. Lillo et al. performed STE analysis of the right atrium and revealed impaired strain values of this structure and all RA strain phases in patients with FD. When considering FD patients without left ventricle hypertrophy (LVH), RA reservoir and contractile strains were signiﬁcantly reduced. The right atrium is therefore another candidate parameter to monitor cardiac TOD in FD.

Indeed, the most advanced technology for the analysis of cardiac structural abnormalities is the MRI, which can provide insight into cardiac hypertrophy features of the Fabry patient. Tondi et al. show that Papillary Muscle hypertrophy is more pronounced in FD, and mitral valve anatomy alterations progressively worsen with advancing FD stages. The findings highlight papillary muscle hypertrophy and mitral valve anatomy abnormalities as potential early markers of cardiac involvement in FD and recommend their routine assessment during cardiac magnetic resonance (CMR) in patients with hypertrophic cardiomyopathies.

Finally, FD often associates with arrhythmias that need to be monitored. To this aim, Roy et al. demonstrate that implantable loop recorders might help to explore the impact of the disease, irrespective of LVH.

## The kidney

Kidney failure is another typical manifestation of the TOD in FD. Rozenfeld et al. focus on Renal fibrosis and consider it as the end result of multiple damages, including inflammation, cell migration, differentiation, and increased extracellular matrix production. In Fabry nephropathy, these effects occur within the kidney tubule, following initial damage caused by lysosomal dysfunction and disruption of Gb3–LysoGb3 activity. Concurrent to mitochondrial failure, kidney tubular cells requiring healthy energetic metabolism to function are the first cells to be damaged in the kidney. Therefore, the contribution of the tubular epithelial cells and the interstitial space to Fabry nephropathy is important from its initiation to its progression and may contribute to the pathogenesis of renal injury.

## Fatigue

Energetic metabolism is also considered a mechanism to fatigue and reduced exercise tolerance in FD, according to De Marco et al. and to Gambardella et al. Fatigue, often an early and independent symptom, can be due to cardiac and pulmonary dysfunction as well as impairments in skeletal muscle. In FD, skeletal muscle bioenergetic alterations such as mitochondrial impairment, metabolic inflexibility, and increased glycolysis might explain the fatigue. Other mechanisms such as inflammation, muscle atrophy, and vascular and neuronal dysfunction further contribute. Cardiopulmonary exercise testing and biomarkers like lactate and mitomiRs might help in the stratification of the clinical condition of patients with FD diagnosis. Enzyme replacement therapy offers limited relief, while personalized exercise programs might offer a more tailored approach to improve patient care and quality of life.

## Inflammation and endothelium

Inflammation is an emerging mechanism for TOD in FD. Reading the review of Kurdi et al., the main activator of inflammation is the accumulation of sphingolipids, resulting from the deﬁciency of alpha galactosidase, which triggers cellular stress. Acute, chronic, and resolving stages are a continuum rather than strictly distinct, although each stage has a recognized hallmark. There are also overlaps between the innate and adaptive immune responses, suggestive of an autoinflammatory component to FD. Inflammation is the possible mechanism behind endothelial dysfunction in FD, which can be monitored through serum levels of VEGF. According to Lund et al. this parameter correlates with markers of renal and cardiac damage in FD patients and can be considered a useful biomarker for endothelial dysfunction.

## Conclusion

Research into FD still has many questions to answer, although it has opened new fields of investigation. The success of this RT shows the need for new papers for “Frontiers in Research topic: Target Organ Damage in Fabry Disease 2.0”.
